# Explainable Recognition of Complex Flight Maneuvers via Retrieval-Augmented Large Language Models

**DOI:** 10.3390/e28080850

**Published:** 2026-07-30

**Authors:** Liqiang Ren, Haipeng Wang, Xinlong Pan, Tiantian Tang, Hongdong Wan

**Affiliations:** 1 Institute of Information Fusion, Naval Aviation University, Yantai 264001, China; hdrlq4492@163.com (L.R.);; 2 National Key Laboratory of Millimeter-Wave and Terahertz Remote Sensing, Yantai 264001, China; 3 Key Laboratory of Sea-Air Information Perception and Processing Technology of Shandong Provincial, Yantai 264001, China; 4 Chinese People’s Liberation Army Unit 92728, Shanghai 200436, China

**Keywords:** complex flight maneuver recognition, large language models (LLMs), table understanding, explainable artificial intelligence (XAI), retrieval-augmented generation

## Abstract

Complex flight maneuver recognition (FMR) underpins intelligent flight training, including training assessment, pilot skill profiling, and flight safety monitoring. Existing FMR methods typically require large labeled datasets, generalize poorly across aircraft, and provide limited decision transparency. We propose TableManeuver, an explainable LLM-based FMR method that reformulates multivariate flight parameter time series as table-understanding inputs. The method updates no base LLM parameters and uses a small labeled training set only as a retrieval library; it is therefore not a zero-shot setting. TableManeuver first converts flight parameter sequences into tabular text that preserves temporal indices and channel semantics, reducing the mismatch between numerical time series and the textual semantic space of LLMs. It then combines domain knowledge, neighborhood sample references, and task decomposition prompts in a retrieval-augmented reasoning architecture that guides explicit step-by-step inference. We evaluate the method on a flight dataset collected from human pilots on a high-fidelity flight simulation platform. Without base LLM parameter updates, TableManeuver achieves 96.2% precision, 96.8% recall, and a 96.5% F1 score, exceeding the strongest supervised baseline by 3.5 percentage points in F1. In cross-aircraft evaluation, the F1 score decreases by only 1.4 percentage points, which is substantially smaller than the degradation observed for deep learning baselines. Retrieval-only baselines that transfer neighbor labels without LLM inference perform markedly worse, indicating that the performance gains are not explained by neighbor label transfer alone. TableManeuver combines recognition accuracy, cross-aircraft robustness, and readable step-by-step reasoning evidence, offering a practical route for applying LLMs to aviation time series analysis.

## 1. Introduction

Driven by the rapid progress of artificial intelligence (AI) and aviation training equipment, the intelligent transformation of flight training has emerged as an important trend in modern aviation training systems, covering automatic assessment of training outcomes, quantitative analysis of pilot proficiency, and emerging applications such as AI flight instructors [1]. As a key enabling technique for these applications, FMR aims to automatically determine the maneuver categories performed by a pilot from multivariate flight parameter time series recorded by onboard sensors (e.g., roll angle, pitch angle, and normal overload). Its recognition performance directly affects the accuracy of training quality assessment and the reliability of pilot skill profiling. However, flight parameter time series exhibit strong temporal dependence, cross-channel coupling, and complex nonlinear characteristics, and are jointly affected by mission profile, aircraft performance, environmental conditions, and individual differences among pilots. Accurately recognizing complex maneuvers directly from raw flight parameters remains a challenging problem [2].

Existing FMR methods can be broadly divided into two categories: expert-knowledge-based rule methods and data-driven learning methods. The former rely on manually constructed recognition rules and parameter thresholds, identifying maneuvers by analyzing variation patterns of altitude, velocity, attitude angles, and related quantities [3,4,5,6,7]. Although such methods are intuitive and interpretable, the construction of rules is costly, and their generalization is limited in the presence of complex maneuvers and pilot-to-pilot variability. Traditional machine learning methods—such as support vector machines [8] and Bayesian networks [9]—introduce a data-driven paradigm and improve recognition performance to some extent, but they typically depend on hand-crafted feature engineering and assume the stationarity of the data, which restricts their effectiveness on dynamic and heterogeneous flight parameter sequences. More recently, deep learning methods have substantially advanced FMR: convolutional neural networks (CNNs) [10,11], recurrent neural networks (RNNs) [12,13,14], Transformer-based architectures [15], and end-to-end temporal detection methods [16] can automatically extract complex features from raw sequences and alleviate the dependence on hand-crafted features. Nevertheless, these methods rely heavily on large-scale labeled data, and the inherent opacity of their decision processes restricts their adoption in real-world scenarios [17].

The rapid development of large language models (LLMs) offers new opportunities to overcome the above limitations. Recent studies have demonstrated that LLMs possess strong capabilities for complex sequence pattern recognition and logical reasoning [18,19], which has motivated the exploration of LLMs for time series analysis [20,21,22]. LLM-based time series methods can be roughly divided into two categories. Prompt-based methods reformulate forecasting or classification tasks as text-to-text generation, allowing LLMs to handle time series problems without parameter tuning [23,24]. Retraining-based methods, in contrast, fine-tune part or all of the LLM parameters to adapt the model to specific time series tasks [25,26]. Since flight parameter sequences are high-dimensional, nonlinear, and multivariate, FMR is essentially a semantic reasoning task that combines temporal pattern analysis with domain knowledge. A natural question arises: can the FMR task be lifted out of the traditional discriminative paradigm and reformulated as an LLM-driven, domain-knowledge-aware generative reasoning task, so as to simultaneously improve recognition performance, reasoning transparency, and cross-aircraft generalization?

Directly applying existing LLM-based methods to FMR, however, still faces several key bottlenecks [27]. (1) Flight parameter sequences are inherently numerical multivariate time series, which are misaligned with the textual semantic space native to LLMs. (2) Existing methods struggle to capture both the dynamic evolution along the temporal dimension and the dependencies along the channel dimension. (3) Task-specific retraining of LLMs is expensive and requires large amounts of labeled data, which limits practicality and cross-aircraft generalization. (4) The capacity of LLMs to perform explicit reasoning grounded in domain knowledge and example references has not yet been fully exploited. These observations indicate the need for a new FMR framework that aligns the numerical and textual modalities efficiently, fully leverages the pretrained knowledge of LLMs, and produces both recognition results and readable reasoning evidence without updating model weights.

Existing LLM-based time series representations fall into three categories, each with its own limitations. Direct text string encoding converts numerical sequences element-by-element into character strings, discarding channel semantics and temporal structure [21]. Embedding-space mapping projects time series data into the latent space of an LLM through an external encoder [25,26], introducing additional trainable parameters and unavoidable information compression. Image-based representation visualizes the time series and feeds the resulting figures into multimodal LLMs [20], sacrificing exact numerical information and failing to support fine-grained value-aware reasoning. In contrast, a tabular representation naturally encodes both the temporal dimension (rows) and the channel dimension (columns), and each numerical cell carries an explicit temporal position and physical semantic annotation. Moreover, tables are a structured data format that LLMs encounter extensively during pretraining, which enables a natural alignment from numerical sequences to the textual semantic space and supports fine-grained reasoning without additional parameter training.

Building on the analysis above, this paper proposes TableManeuver, an explainable recognition framework that recasts FMR as an LLM-driven table-understanding task. The framework requires no fine-tuning or parameter updates of the base LLM. It uses a small labeled training set only as a retrieval library, and is therefore not a zero-shot method. First, tabular encoding reformats multivariate flight parameter sequences with time steps as rows and parameter channels as columns. This representation preserves temporal continuity and channel semantics while aligning numerical sequences with the semantic space of LLMs. Second, a knowledge–task dual-driven retrieval-augmented reasoning architecture integrates contextual domain knowledge, neighborhood retrieval, and task decomposition prompts to guide explicit step-by-step reasoning. By combining these strategies, TableManeuver recognizes complex flight maneuvers without updating model parameters and generates an interpretable reasoning trace. The main contributions are as follows.

1. We propose a tuning-free FMR paradigm that requires no base LLM parameter updates and reformulates FMR as a table-understanding task. The tabular flight parameter representation preserves both temporal and channel dimensions, improving structural fidelity and numerical–semantic expressiveness relative to existing LLM-based time series encodings.
2. We design a knowledge–task dual-driven retrieval-augmented reasoning framework. By integrating domain knowledge, neighborhood retrieval, and task decomposition prompts, the framework guides the LLM to recognize complex flight maneuvers without updating base model parameters while producing a structured reasoning process.
3. We conduct comprehensive experiments on a flight dataset collected from human pilots on a high-fidelity simulation platform. The results show that TableManeuver outperforms supervised baselines in recognition performance, interpretability, and cross-aircraft generalization, supporting the effectiveness of the proposed paradigm.

The remainder of this paper is organized as follows. Section 2 reviews related work. Section 3 describes the core components of the TableManeuver framework in detail. Section 4 presents the experimental results and analyses from multiple perspectives. Section 5 concludes the paper and outlines future work.

## 2. Related Work

### 2.1. Complex Flight Maneuver Recognition

Research on FMR can be broadly divided into two stages: expert-knowledge-driven methods and data-driven methods. Early approaches relied heavily on domain expert knowledge, performing recognition through manually constructed rule bases or knowledge bases. For instance, Ni et al. [3] built an aircraft flight parameter knowledge base for the recognition of complex maneuvers; Wang et al. [4] developed a load analysis program that examines the numerical ranges and shape features of parameter curves; and Meng et al. [5] attempted to recognize complex maneuvers through the logical combination of basic maneuvers. These methods exhibit clear logic and strong interpretability, but their robustness is limited when handling non-standard or complex maneuvers, and the cost of building and maintaining their rule bases is considerable.

With the rise of the data-driven paradigm, the recognition performance and application scope of FMR have been greatly expanded. In the early data-driven studies, Yang et al. [8] proposed a flight action recognition method based on fuzzy support vector machines, and Shen et al. [9] modeled probabilistic dependencies among flight actions using Bayesian networks. In recent years, deep learning models have become mainstream by directly learning temporal patterns from data. Fang et al. [10] and Zhu et al. [11] designed local feature extraction structures based on CNNs; Zhou et al. [12] and Fang et al. [14] introduced RNNs to model temporal dependencies; Li et al. [13] proposed a focused-attention BiLSTM to address the difficulty of modeling long-range temporal features; Ren et al. [15] further introduced a lightweight CNN–Transformer hybrid network built on wavelet time–frequency maps to more effectively model both global and channel-wise interactions; and Wang et al. [16] proposed FM-detector, an end-to-end temporal detection method that advances the automation and complex-maneuver recognition capability to a new level. Although these methods have made significant progress in accuracy, their black-box nature leaves the decision process opaque, they remain heavily dependent on large-scale high-quality labeled data, and they cannot easily incorporate domain knowledge expressed in natural language.

### 2.2. Large Language Models for Time Series Analysis

The recent success of LLMs in natural-language processing has motivated their application to time series analysis [28]. Retraining-based methods fine-tune pretrained LLMs on specific time series datasets to adapt them to target tasks. For example, Time-LLM [25] reprograms time series data into the input space of an LLM; CrossTimeNet [29] adopts a cross-domain pretraining strategy to learn transferable features; GPT4TS [30] explores the general time series analysis ability of pretrained language models; and Multimodal-LLM [26] investigates multimodal fusion mechanisms. However, such methods require large amounts of labeled data and substantial computational resources, which limits their practical use. Prompt-based methods, by contrast, directly exploit the pretrained knowledge and reasoning ability of LLMs and guide them with carefully designed prompts. For instance, LLMTime [21] encodes numerical time series directly as text strings and leverages the text completion mechanism to generate predictions; PromptCast [24] reformulates time series forecasting as a prompt-based sentence-to-sentence task; and TimeReasoner [31] further taps into the slow-thinking reasoning capability of LLMs by generating explicit reasoning traces to analyze the intrinsic dynamics of time series. These studies provide the theoretical foundation and methodological inspiration for TableManeuver. Building on them, this paper investigates how to adapt LLMs to FMR over flight parameter time series so as to achieve both interpretable and high-accuracy recognition.

## 3. The TableManeuver Framework

This section first gives the problem formulation, and then presents the design of each core component of TableManeuver together with the complete algorithmic procedure.

### 3.1. Problem Formulation

Let the flight maneuver dataset be denoted as $D=\left\{\left(X^{1},y^{1}\right),\left(X^{2},y^{2}\right),\ldots ,\left(X^{n},y^{n}\right)\right\}$, where each sample $X^{i}\in {\mathbb{R}}^{t\times m}$ is a multivariate time series of t time steps and m parameter channels, and $y^{i}\in \left\{c_{1},c_{2},\ldots ,c_{k}\right\}$ is the corresponding maneuver category label. Traditional FMR methods aim to learn a discriminative model on D that maps the input space X to a probability distribution over the category y.

In TableManeuver, by combining task characteristics with prompt engineering, the FMR problem is reformulated as a table-understanding problem. Let P denote a structured prompt designed for time series inputs and the FMR task. Given a sample $X^{i}$ to be recognized, the model output is formalized as

(1) $$T^{i}=\mathrm{LLM}(P,X^{i})$$

where $T^{i}$ is the textual response generated by the LLM under the prompt P and the tabular time series input $X^{i}$, and contains both the predicted category and an interpretable reasoning process. The text $T^{i}$ is then post-processed (e.g., through keyword-based discrimination or structured parsing) to extract the predicted label ${\hat{y}}^{i}$ and the reasoning explanation ${\hat{E}}^{i}$, completing the recognition of the flight maneuver category together with the generation of its decision evidence.

(2) $$P(X^{i})=\mathrm{Concat}(P_{\mathrm{ctx}},\;P_{\mathrm{nbr}}(X^{i}),\;X_{\mathrm{text}}^{i},\;P_{\mathrm{dec}})$$

where $P_{\mathrm{ctx}}$ denotes contextual information that encodes task-specific domain knowledge. $P_{\mathrm{nbr}}(X^{i})$ is the neighborhood-augmented context retrieved for $X^{i}$, including positive and negative labeled references. $X_{\mathrm{text}}^{i}$ is the serialized tabular representation of the test sample, and $P_{\mathrm{dec}}$ is the task decomposition instruction that defines the three-step reasoning protocol and output format. The Concat operator denotes ordered textual concatenation. Among these components, $P_{\mathrm{ctx}}$ and $P_{\mathrm{dec}}$ are shared across samples, whereas $P_{\mathrm{nbr}}(X^{i})$ and $X_{\mathrm{text}}^{i}$ are instance-dependent. Substituting Equation (2) into Equation (1) shows that TableManeuver conditions a frozen LLM on retrieval- and knowledge-augmented context rather than modifying its parameters.

### 3.2. Overall Framework

**Figure 1:**
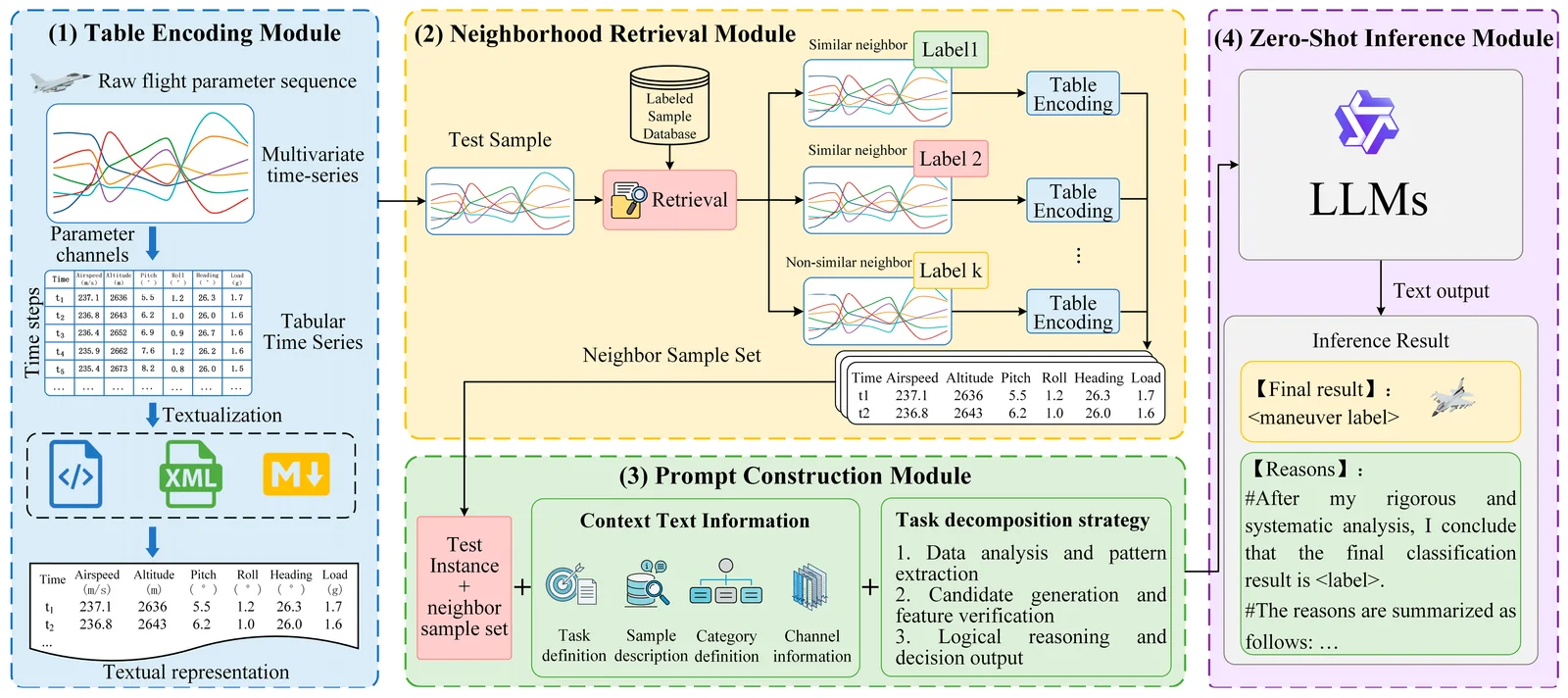
Overall architecture of the TableManeuver framework.

As illustrated in Figure 1, TableManeuver comprises four core components. First, the neighborhood retrieval module selects positive and negative reference samples relevant to the test sample from a small labeled training set, providing instance-level contextual information. Second, the tabular-encoding module transforms raw numerical flight parameter sequences into a structured tabular representation that preserves both temporal and channel information. Third, the prompt-construction module integrates domain context, retrieved neighborhood samples, and task decomposition instructions into a structured prompt for LLM reasoning. Fourth, the tuning-free reasoning module predicts the maneuver category and generates a textual explanation based on the constructed prompt.

### 3.3. Tabular Encoding and Textual Representation of Flight Parameter Sequences

#### 3.3.1. Tabular Encoding Strategy

Existing LLM-based time series methods typically learn time series embeddings directly in the latent space of an LLM, or rely on an external encoder to map the resulting embeddings into the LLM’s representation space. However, such indirect mapping often incurs information loss and struggles to fully preserve the temporal dependencies and the physical semantics and statistical characteristics specific to each parameter channel. At the same time, the modality gap between numerical flight parameter sequences and the native textual semantic space of LLMs further restricts the ability of LLMs to capture and understand the complex patterns and key features of flight parameter data.

To address these issues, this paper proposes a tabular encoding strategy that reformats multivariate flight parameter time series into tabular data. In the table, rows correspond to time steps and columns correspond to parameter channels, so that each numerical cell simultaneously carries a temporal-position label and a physical semantic label. This representation fully preserves the temporal and channel structure of the original sequences while being naturally compatible with the structured data formats widely seen by LLMs during pretraining, enabling a natural alignment from numerical sequences to the textual semantic space.

Let the input feature space of the tabular encoding module be $X_{i}\in {\mathbb{R}}^{L_{i}\times m}$, where $L_{i}$ is the number of time steps of the i−th sample and m is the number of parameter channels. By adding a column name vector $C^{⊤}=(c_{1},c_{2},\ldots ,c_{m})$ and a temporal index $T={\left(t_{1},t_{2},\ldots ,t_{L_{i}}\right)}^{⊤}$, the data are reorganized into the tabular form ${X^{\prime }}_{i}$:

(3) $${X^{\prime }}_{i}=\left(\begin{array}{ccccc} 0 & c_{1} & c_{2} & \ldots & c_{m}\\ t_{1} & x_{1,1} & x_{1,2} & \ldots & x_{1,m}\\ t_{2} & x_{2,1} & x_{2,2} & \ldots & x_{2,m}\\ \vdots & \vdots & \vdots & \ddots & \vdots \\ t_{L_{i}} & x_{L_{i},1} & x_{L_{i},2} & \ldots & x_{L_{i},m} \end{array}\right)\;$$

where $C^{⊤}$ contains the parameter names corresponding to each channel (e.g., airspeed, altitude, pitch angle, roll angle, heading angle, and normal overload), T is the temporal index, and $X_{i}$ is the original multivariate flight parameter time series. The tabular flight parameter data are then serialized into a textual representation suitable as input to the LLM:

(4) $$X_{\mathrm{text},i}=\mathrm{Serialize}(X_{i}^{\prime })$$

where $X_{\mathrm{text},i}$ denotes the serialized textual representation and Serialize(⋅) is the serialization function. Several textual formats are supported, including Markdown, HTML, JSON, and DFLoader [32]. Different encoding formats differ in token consumption and semantic clarity, as analyzed in detail in Section 4.6.4.

#### 3.3.2. Implementation Details of the Encoding

To balance inference efficiency and feature fidelity, the following encoding conventions are adopted.

(1) Preservation of original physical scales. To retain the physical semantics of each variable, the proposed method does not normalize the input features and instead uses values in their original units, including velocity (m/s), altitude (m), angle (°), and overload (g). This strategy allows the LLM to make use of the physical common sense acquired during pretraining. For example, when the LLM observes a normal overload of 3.8 g, it can directly associate this value with prior knowledge about high-intensity maneuvers.

(2) Fixed-rate down-sampling. Because of framerate fluctuations at the data acquisition interface, the raw flight parameter sequences have uneven sampling intervals and considerable lengths, and directly encoding them as text would consume excessive tokens. We therefore adopt a nearest-neighbor resampling strategy: each one-second interval is divided into $f_{t}$ sub-windows according to the target frame rate $f_{t}$, with each sub-window of width $\Delta w=1/f_{t}$ seconds. Within each sub-window, the original sample point nearest to the window center is selected as the representative value, with no interpolation, so that every retained value is an original record.

(3) Unified numerical precision. All values are uniformly retained to one decimal place. This both maintains the distinguishability of the physical features and reduces the token overhead caused by redundant precision, while improving the readability of the tabular text.

(4) DFLoader encoding format. We adopt the DFLoader format [32], which represents tabular data as column-aligned plain text, with row indices given as actual timestamps and column headers carrying parameter names and units. Compared with HTML and JSON, DFLoader introduces no redundant markup symbols and offers higher token efficiency; the experimental evidence is presented in Section 4.6.4.

### 3.4. Construction of Contextual Textual Information

LLMs inherently lack task-specific knowledge and rely heavily on explicit prompts to steer their reasoning. To alleviate this issue, we introduce a domain context as a core component of the prompt, which explicitly supplies task-specific knowledge and relevant background information.

The domain context is generated according to a structured template, whose key components are as follows. (1) Task definition: a clear description of the task objective, namely, recognizing maneuver categories from flight parameter time series. (2) Dataset description: an explanation of the dataset characteristics, including the aircraft types involved, the temporal length, the parameter dimensionality, and the sampling frequency. (3) Category definitions: detailed explanations of the meaning of each category label, including the standard definition of each maneuver, its discriminative features, and typical parameter variation patterns. (4) Channel information: descriptions of the physical meaning, numerical range, and role in recognition of each parameter channel. This structured template provides the LLM with a complete task context, enabling its reasoning to take place under explicit domain constraints.

### 3.5. Neighborhood-Augmented Reasoning

Without task-specific examples, LLMs find it difficult to reliably distinguish maneuver categories that are highly similar to one another. To address this issue, we introduce a neighborhood-augmented reasoning mechanism that provides the LLM with critical contextual guidance by retrieving relevant neighborhood samples from labeled training data. These neighborhood samples include positive examples that share key features with the test sample and negative examples that exhibit contrasting patterns; together they serve as reliable references that help the model produce more accurate predictions.

Specifically, two neighborhood retrieval strategies are used: positive-only retrieval and contrastive-augmented retrieval. For the former, a similarity measure is used to identify the k nearest-neighbor samples for each test sample from a small set of labeled training data. To prevent large-scale channels such as altitude and velocity from dominating the distance computation, z-score normalization based on training-set statistics is applied only to the flight parameter sequences used in similarity computation during the retrieval stage; the tabular text fed into the LLM still retains the original physical scales, so that the numerical semantics are preserved. The retrieval process is formalized as

(5) $$\mathrm{Retrieval}(X_{\mathrm{test}},D_{\mathrm{train}},d,k)=\underset{k}{\arg\min}{\left[d(X_{\mathrm{test}},X_{i})\right]}_{i=1}^{\left|D_{\mathrm{train}}\right|}\;$$

where $X_{\mathrm{test}}$ denotes the test sample, $D_{\mathrm{train}}$ the training set, d the similarity measure used, and k the desired number of neighbors. The retrieval library is constructed exclusively from labeled training samples; in cross-aircraft testing, no label information of the target aircraft is introduced.

To further improve the model’s ability to discriminate between adjacent category boundaries, a contrastive-augmented retrieval mechanism is introduced to incorporate negative-sample references during reasoning. First, the training set is clustered using K-means. Then, for each test sample, the cluster containing its positive samples is identified, and representative negative samples are randomly drawn from other clusters that do not contain positive samples. The K-means clustering process is formalized as

(6) $${\left\{C_{k}\right\}}_{k=1}^{K}=\underset{{\left\{C_{k}\right\}}_{k=1}^{K}}{\arg\min}\sum_{k=1}^{K}\sum_{x_{i}\in C_{k}}\|x_{i}-\mu_{k}{\|}^{2}\;$$

where $C_{k}$ is the k−th cluster, $\mu_{k}$ is its centroid, and K is the number of clusters. We set K equal to the number of categories (K=9), so that each cluster approximately corresponds to one maneuver category. For each test sample, its k nearest neighbors are first retrieved as positive samples through Equation (5), and the clusters to which these positive samples belong are identified; negative samples are then randomly drawn from the remaining clusters that do not contain any positive sample. By introducing these negative samples, TableManeuver leverages contrastive information to help the LLM better discriminate among categories.

The supervised retrieval in Equation (5) and the clustering in Equation (6) serve distinct roles. Equation (5) selects the k nearest labeled training samples as positive references. Equation (6) is used only for negative-sample sourcing. Specifically, K-means partitions the training set without labels, and negatives are drawn from clusters that do not contain the positive neighbors. Thus, retrieval provides precise positive evidence, whereas clustering provides diverse contrastive evidence; clustering never assigns the final label.

The choice of K-means over alternative clustering techniques follows from this auxiliary role. Because the partition is used only to source negatives, the requirement is a complete, coarse, and computationally cheap partition rather than a maximally accurate one. Density-based methods such as DBSCAN require density hyperparameters and may designate samples as noise, excluding them from every cluster and hence from the negative pool. Hierarchical agglomerative clustering incurs at least quadratic cost in the library size and still requires a cut-off selection, and Gaussian mixture models add covariance estimation without benefit, since cluster quality is not critical here. K-means yields a complete partition at linear cost per iteration with a single interpretable hyperparameter K, and because clustering only diversifies the negative pool, the framework is insensitive to the specific clustering algorithm employed.

Negatives are drawn randomly from the non-positive clusters, rather than by hard-negative mining, for two reasons. First, the purpose of the negative reference is to provide a clear, unambiguous contrast; the hardest negatives are boundary samples that are themselves easily confusable and may mislead the elimination step. Second, random sampling is unbiased and adds no extra distance computation. The ablation study in Section 4.6.5 shows that a single randomly drawn negative already yields the peak performance, supporting this design.

We set K equal to the number of maneuver categories K=9 as a practical default for negative sourcing. K is decoupled from retrieval and from the final LLM decision, so clusters need not match categories exactly. For a dataset with C classes, K=C is a natural default, but the framework can also use a moderate constant K without changing the retrieval or reasoning modules. Neighborhood samples and contextual information provide complementary evidence. The former offers instance-level pattern references, whereas the latter grounds the reasoning in maneuver definitions and physical semantics.

### 3.6. Structured Prompt Design

#### 3.6.1. Task Decomposition

Traditional prompts often lack structured guidance, which exposes LLMs to difficulties when handling complex tasks. Without explicit task instructions, the model must infer the task objective and execution path on its own, which can easily lead to misinterpretation of semantics, inconsistent reasoning, or outputs that drift away from the task requirements. To mitigate this problem, we propose a task decomposition mechanism that breaks down the complex FMR task into a sequence of fine-grained, logically progressive sub-steps. Specifically, the FMR task is decomposed into the following three steps.

(1) Data parsing and pattern extraction. The variation trends, inter-channel relationships, and temporal characteristics of the main parameters are parsed; key phases are identified; and discriminative temporal patterns are distilled.

(2) Candidate generation and feature verification. The extracted patterns are compared against the domain knowledge base and the neighborhood samples to generate candidate categories, whose discriminative physical features are then verified.

(3) Logical reasoning and decision output. On the basis of strict logic, candidates are falsified step by step and inconsistent ones are eliminated, after which the maneuver category with the highest confidence and its reasoning process are output.

This three-step strategy helps the LLM converge progressively to a more accurate recognition result, improving both decision quality and interpretability.

#### 3.6.2. Complete Prompt Template

**Figure 2:**
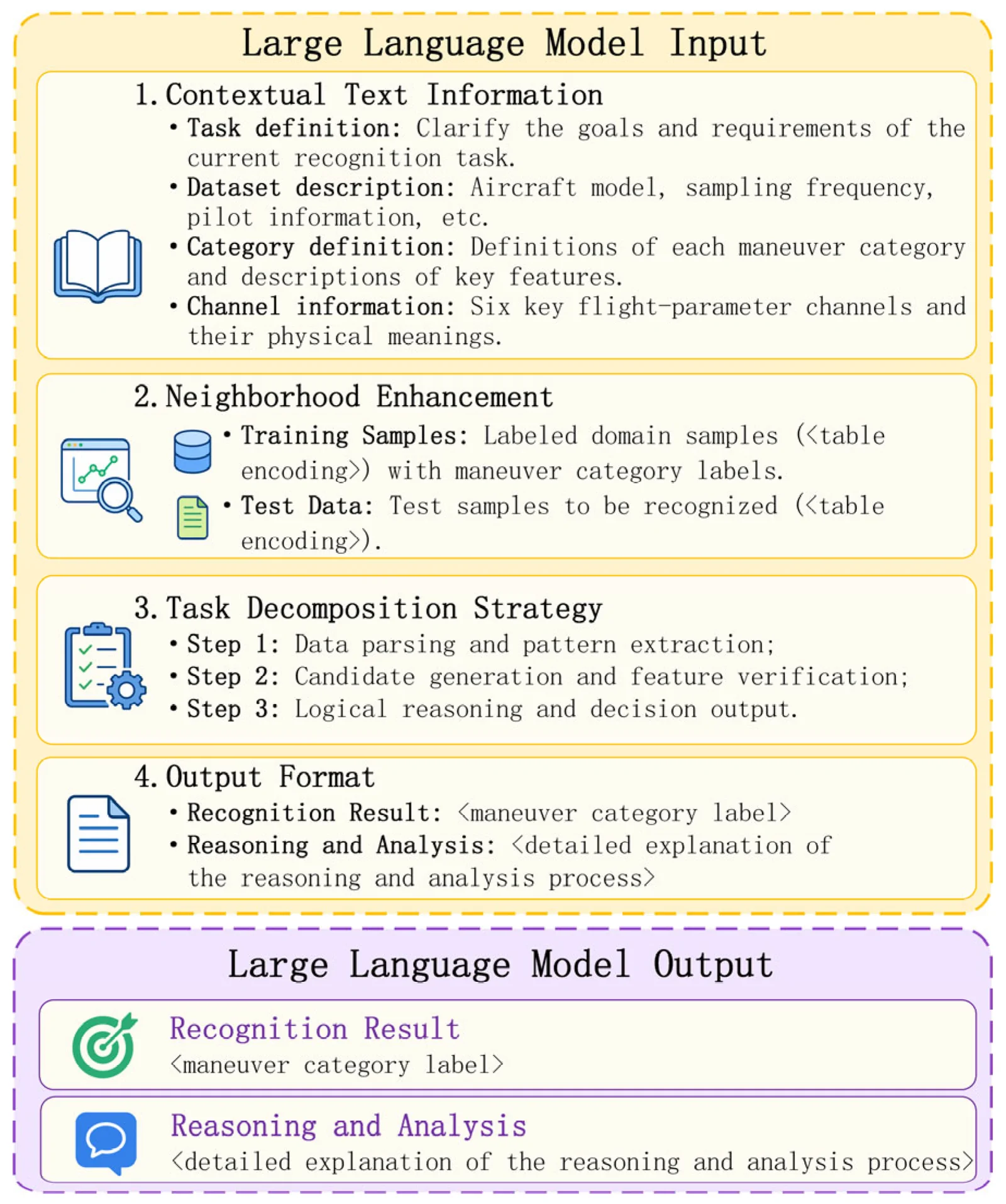
The prompt template of TableManeuver.

In TableManeuver, the structured prompt is the key enabler of tuning-free reasoning. The contextual textual information provides specialized domain knowledge for the LLM; the neighborhood samples provide data-driven context; and the task decomposition mechanism guides the LLM to perform step-by-step reasoning. The complete prompt template is shown in Figure 2.

### 3.7. Algorithm Procedure

Building upon the modules introduced above, Algorithm 1 presents the complete procedure of TableManeuver in pseudocode to facilitate reproducibility.
**Algorithm 1: TableManeuver Flight Maneuver Recognition**$\mathrm{Input}:\;\mathrm{test}\;\mathrm{sample}\;X_{\mathrm{test}}\in {\mathbb{R}}^{t\times m}$; $\mathrm{training}\;\mathrm{set}\;D_{\mathrm{train}}$; pretrained LLM M$;\;\mathrm{raw}\;\mathrm{frame}\;\mathrm{rate}\;f_{s}$$;\;\mathrm{target}\;\mathrm{frame}\;\mathrm{rate}\;f_{t}$; number of positive samples k$;\;\mathrm{number}\;\mathrm{of}\;\mathrm{negative}\;\mathrm{samples}\;k_{neg}$; number of clusters K.**Output**: predicted category $\hat{y}$ reasoning explanation $\hat{E}$.1. **Stage 1: Tabular encoding (fixed-rate down-sampling)**2. $\Delta w\leftarrow 1/f_{t}$, width of a single sub-window3. **for each** one-second interval [*s*, *s* +1) **do**4.   Divide [s,s+1)$\;\mathrm{into}\;f_{t}$ sub-windows5.   **for each** sub-window j **do**6.     $t^{c}\leftarrow s+(j+0.5)\Delta w$
7.     
$\tilde{x}\leftarrow X_{\mathrm{test}}[\arg\min_{i:t_{i}\in \mathrm{sub}\text{-}\mathrm{window}}|t_{i}-t^{c}|]$
8.   **end for**9. **end for**10. $X_{\mathrm{test}}\leftarrow [{\tilde{x}}_{0},{\tilde{x}}_{1},\ldots ]$, down-sampled sequence11. ${X^{\prime }}_{\mathrm{test}}\leftarrow \mathrm{TableEncode}(X_{\mathrm{test}},C,T)$, according to Equation (3)12. $X_{\mathrm{text}}\leftarrow \mathrm{Serialize}({X^{\prime }}_{\mathrm{test}},\mathrm{DFLoader})$, according to Equation (4)13. **Stage 2: Neighborhood retrieval**14. $N^{+}\leftarrow \mathrm{Retrieval}(X_{\mathrm{test}},D_{\mathrm{train}},\mathrm{DTW},k)$, according to Equation (5)15. ${\left\{C_{j}\right\}}_{j=1}^{K}\leftarrow \mathrm{KMeans}(D_{\mathrm{train}},K)$, according to Equation (6)16. 
$N^{-}\leftarrow \mathrm{Sample}\mathrm{Negative}(N^{+},\{C_{j}\},k_{neg})$
17. 
$\mathrm{Apply}\;\mathrm{tabular}\;\mathrm{encoding}\;\mathrm{to}\;\mathrm{each}\;\mathrm{sample}\;\mathrm{in}\;N^{+}$
$\;\mathrm{and}\;N^{-}$
18. **Stage 3: Comprehensive prompt construction**19. 
$P_{\mathrm{context}}\leftarrow \mathrm{Build}\mathrm{Context}(\mathrm{task}\;\mathrm{definition},\;\mathrm{dataset}\;\mathrm{description},\;\mathrm{category}\;\mathrm{definitions},\;\mathrm{channel}\;\mathrm{information})$
20. 
$P_{\mathrm{neighbor}}\leftarrow \mathrm{BuildNeighbor}(N_{\mathrm{text}}^{+},N_{\mathrm{text}}^{-})$
21. 
$P_{\mathrm{decomp}}\leftarrow [\mathrm{data}\;\mathrm{parsing}\to \mathrm{candidate}\;\mathrm{verification}\to \mathrm{logical}\;\mathrm{decision}]$
22. 
$P\leftarrow \mathrm{Concat}(P_{\mathrm{context}},P_{\mathrm{neighbor}},X_{\mathrm{text}},P_{\mathrm{decomp}})$
23. **Stage 4: Tuning-free inference**24. 
T←M(P)
25. 
$\hat{y},\;\hat{E}\leftarrow \mathrm{Parse}(T)$
26. 
$\mathrm{return}\;\hat{y},\;\hat{E}$


We further analyze the complexity of Algorithm 1. Let L denote the down-sampled sequence length, m the number of channels, N the size of the retrieval library, and Lp and Lg the prompt and output lengths in tokens. Tabular encoding costs O(Lm) time. DTW between two sequences costs O(L^2^ m) time and O(L^2^) space, reducible to O(wLm) and O(wL) with a Sakoe-Chiba band of width w, so retrieval over the library costs O(NL^2^ m) per query and scales linearly with the library size; for substantially larger libraries, lower-bound pruning (e.g., LB_Keogh), constrained or approximate DTW, and cluster-based candidate pruning reduce the number of exact DTW computations without altering the framework. K-means clustering is a one-time offline cost amortized over all queries. LLM inference costs O(Lp^2^) for prefilling and O(Lg·Lp) for autoregressive decoding, with memory dominated by the model weights and a KV cache that grows linearly in Lp + Lg. The measured runtime in Section 4.3 (approximately 1.8 s for retrieval versus 38 s end-to-end per sample) confirms that LLM inference dominates the overall cost.

## 4. Experimental Results and Analyses

To assess the effectiveness of the TableManeuver framework, this section first systematically evaluates the recognition performance and cross-aircraft generalization of the proposed method on a self-built high-fidelity simulated flight dataset. Then, ablation studies and retrieval strategy analyses examine the contribution of each core component, and additional experiments on cross-LLM adaptability, representative case studies, and human-based interpretability evaluation verify the practicality and explainability of the method from multiple perspectives.

### 4.1. Dataset

To date, no publicly available standard benchmark dataset exists in the FMR field. To systematically evaluate TableManeuver, we build a flight maneuver dataset that covers multiple types of typical complex maneuvers, multiple participants, and multiple aircraft platforms, using the high-fidelity digital flight simulation platform DCS World. The platform records the continuous state evolution of the aircraft during maneuvers and retains key flight parameters such as velocity, altitude, attitude angles, and overload, thus providing physically meaningful temporal samples for FMR.

**Figure 3:**
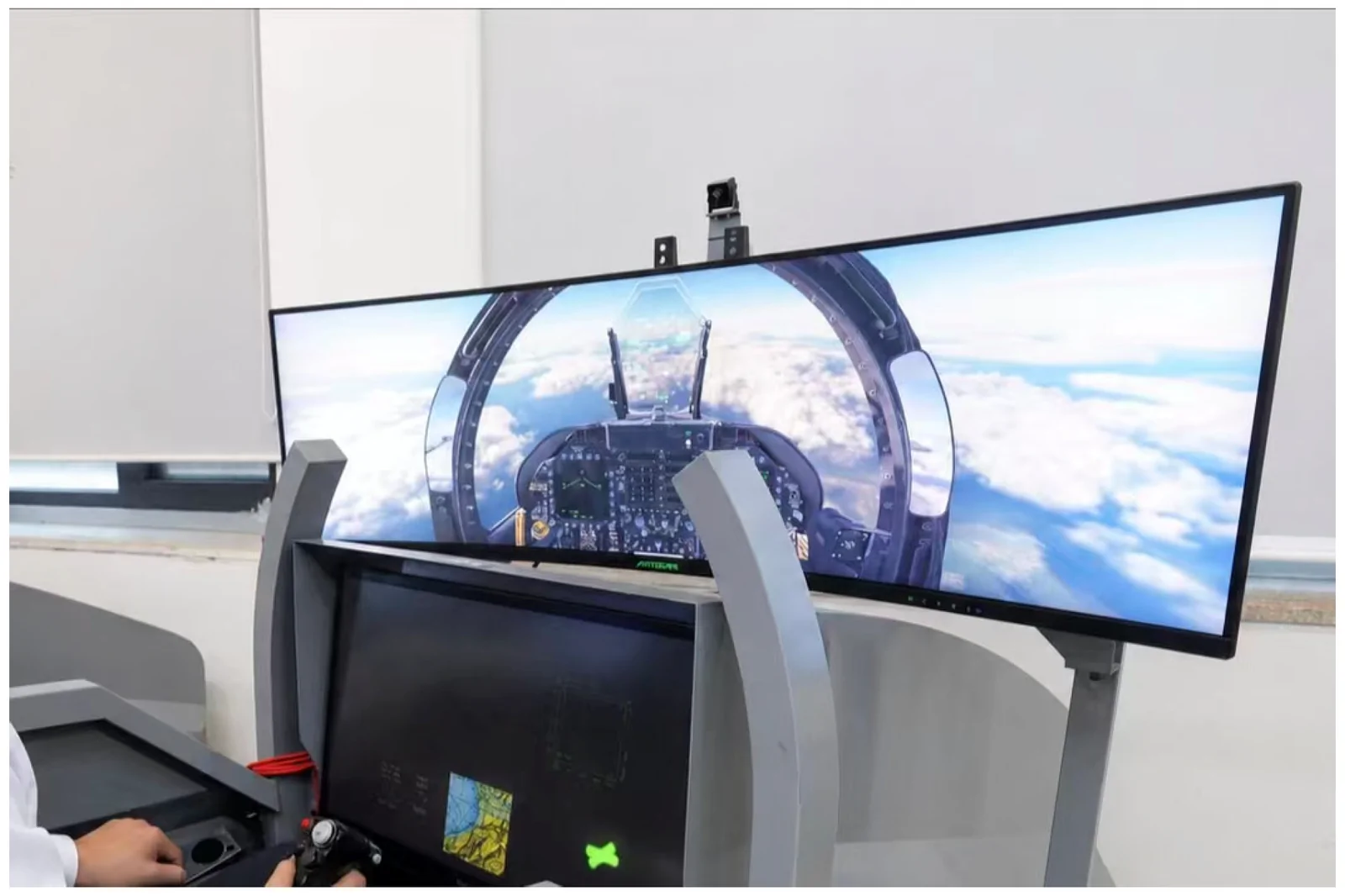
Data acquisition experimental environment.

The dataset contains nine categories of typical complex flight maneuvers: steep turn, aileron roll, Split-S, loop, half-loop with roll, cloverleaf loop, climbing sharp turn, descending sharp turn, and Half Cuban Eight. Each sample consists of six key flight parameter channels, namely air speed, altitude, pitch angle, roll angle, heading angle, and normal overload. Among them, air speed and altitude reflect the energy state of the aircraft; pitch, roll, and heading angles describe spatial attitude changes; and normal overload characterizes the intensity of the maneuver. Together these channels form the discriminative feature space for FMR. Figure 3 illustrates the data acquisition environment. The pilots completed maneuvers in a cockpit-style simulator following standardized maneuver definitions, while the system synchronously recorded flight parameter sequences and process information.

**Figure 4:**
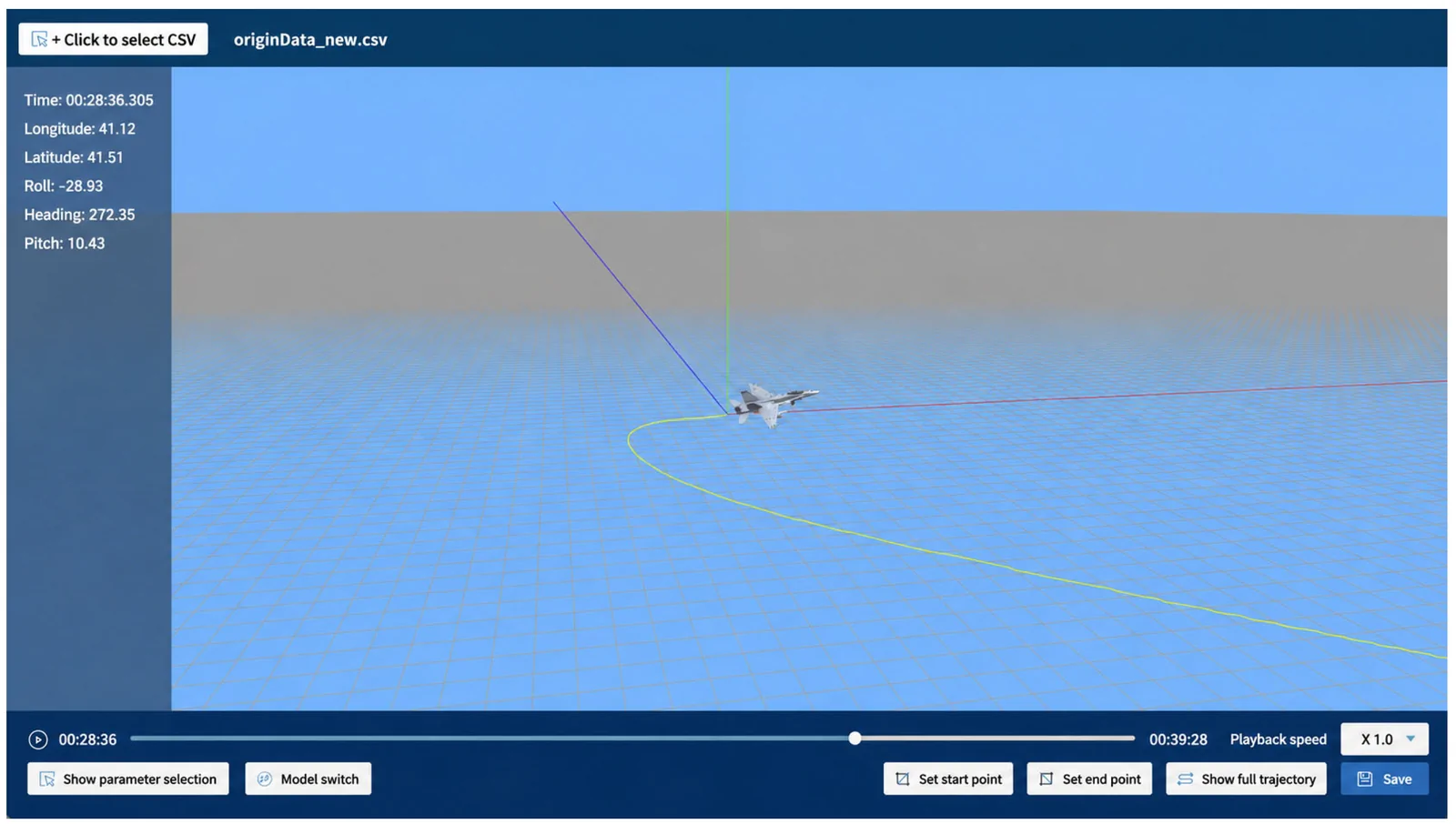
Interface of flight maneuver annotation and 3D trajectory visualization tool.

A total of 25 pilots participated in data collection, including 20 trainee pilots, each with more than 50 h of cumulative flight time, and 5 senior flight instructors, each with more than 500 h of cumulative flight time. Each participant independently performed the maneuvers on the simulator according to standardized maneuver definitions. To ensure annotation quality, a three-level review mechanism is adopted. First, the pilot who executed the flight task performs an initial annotation of his or her own data immediately after the flight. Next, domain specialists conduct a secondary review and re-annotation of the initial labels. Finally, senior flight instructors perform a final confirmation. The entire annotation workflow is supported by an in-house flight maneuver annotation tool that provides synchronized display of flight parameters, 3D visualization of trajectory and attitude, and segment-cutting functionality. The main interface is shown in Figure 4.

**Figure 5:**
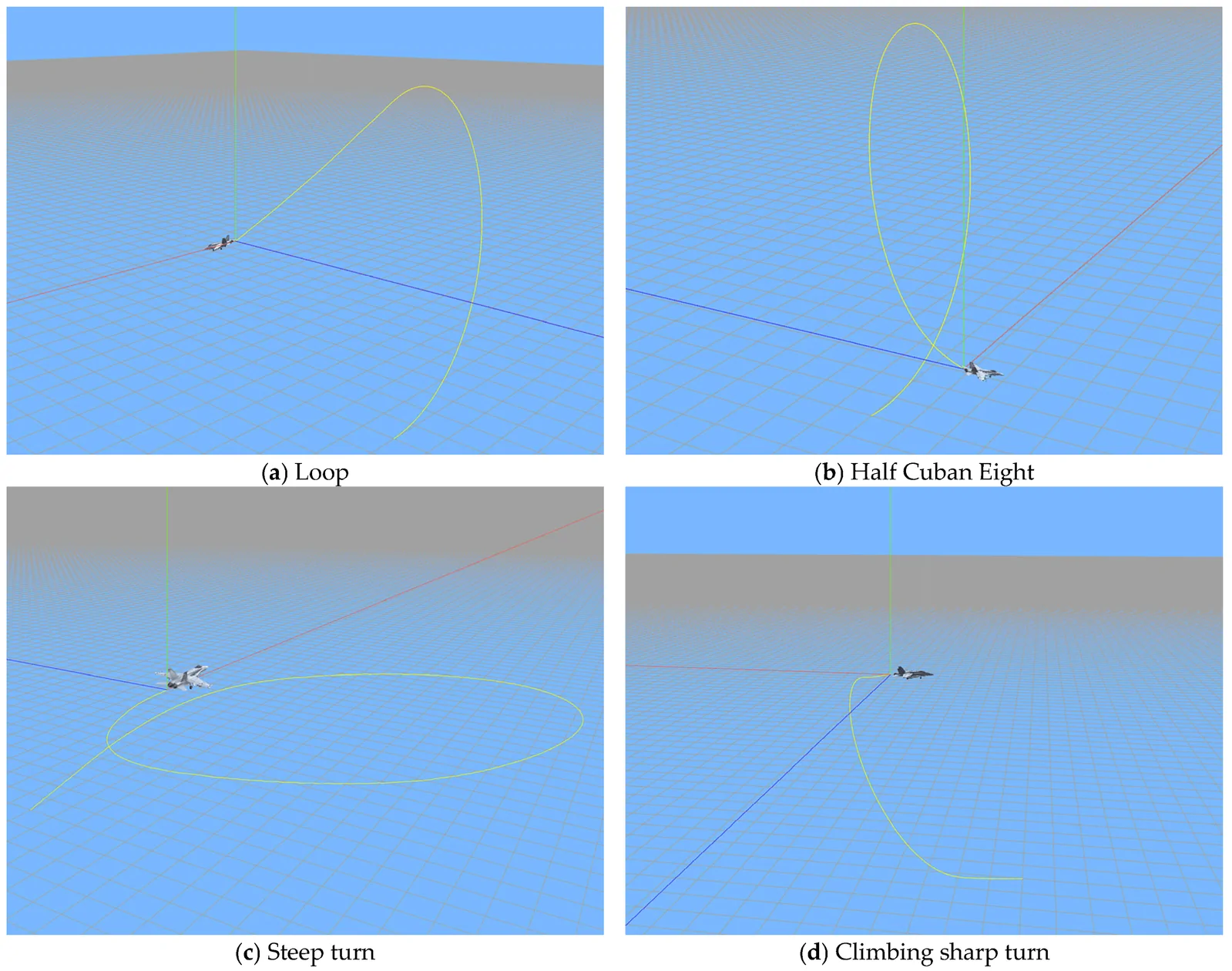
3D trajectory examples of representative complex flight maneuvers: (**a**) Loop; (**b**) Half Cuban Eight; (**c**) Steep turn; (**d**) Climbing sharp turn.

To intuitively illustrate the morphological differences among categories, Figure 5 shows 3D trajectory examples of four representative maneuvers: (a) Loop; (b) Half Cuban Eight; (c) Steep turn; (d) Climbing sharp turn.

The experiments adopt two representative high-performance aircraft platforms, F-18 and F-16. The dataset is split according to a pilot-independent principle into an F-18 training set, an F-18 test set, and a cross-aircraft F-16 test set, where the pilots in the training set and in the F-18 test set do not overlap. The F-16 test set is used neither for model training nor for hyperparameter selection nor for neighborhood library construction; it is used solely for evaluating cross-aircraft generalization. TableManeuver uses the F-18 training set as the labeled retrieval library in both F-18 and F-16 testing, to avoid leakage of label information from the target aircraft. Table 1 summarizes the main statistics of the dataset.

### 4.2. Baselines

To comprehensively evaluate the recognition performance of TableManeuver, we compare it with eight representative baselines in four categories: traditional methods, retrieval-only methods, deep learning methods, and LLM-driven methods. The traditional baseline is MACTW [33]. The retrieval-only baselines include 1-NN (DTW) label transfer and kNN (DTW, k=3) majority vote, which use the same retrieval library and distance function as TableManeuver but assign labels directly from retrieved samples without invoking an LLM. The deep learning baselines include CNN [11], LSTM [13], and ConvBiFormer [15], and the LLM-driven baselines are Time-LLM [25] and TimeReasoner [31]. For fairness, all LLM-based baselines receive the same task description and category definitions as TableManeuver, and all deep learning baselines use the same down-sampled flight parameter sequences as input, follow the hyperparameters recommended in their original papers, and are trained on the training set until convergence. Except for TimeReasoner, MACTW, and the two retrieval-only baselines, all baselines require supervised training. In contrast, TableManeuver updates no LLM parameters and retrieves only a small number of labeled training samples as contextual references. In this setting, labeled samples are reused only as retrieval references, and no base model parameter is updated.

### 4.3. Implementation Details

Experiments are conducted on a workstation equipped with a single NVIDIA RTX 4090 GPU with 24 GB of memory. The software environment consists of Python 3.10, PyTorch 2.5.1, and CUDA 12.4. Each baseline follows the hyperparameters recommended in its original paper and, when training is required, is trained on the training set until convergence. By default, TableManeuver uses Qwen3.6-35B-A3B as the base LLM and updates no model parameters during inference. Tabular encoding adopts the DFLoader format, while neighborhood retrieval uses DTW as the distance function, with k=3 positive samples, $k_{\mathrm{neg}}=1$ negative sample, and k=9 clusters.

For reproducibility, Qwen3.6-35B-A3B is deployed using vLLM with AWQ 4-bit weight quantization (W4A16). Although the model contains 35B parameters in total, only approximately 3B parameters are activated per token, allowing inference to fit within the 24 GB memory of a single RTX 4090. The quantized weights occupy approximately 18.5 GB, and the peak memory usage is approximately 22.6 GB. The maximum context length is set to 40,960 tokens. Across the test set, the actual prompt length averages approximately 6.8K tokens, while the generated reasoning trace averages approximately 1.3K tokens. Generation is performed with temperature T=0 and a batch size of 1. The average end-to-end processing time is approximately 38 s per sample, of which DTW retrieval accounts for approximately 1.8 s. Complete runnable prompts are released in the public repository. Each experiment is repeated five times using different random seeds. For TableManeuver, the random seeds affect random negative sampling and K-means initialization. Deterministic methods, including MACTW and the two retrieval-only baselines, produce identical results across repeated runs and are reported without standard deviations. To assess whether the observed improvements are statistically significant, we apply Welch’s two-sample *t*-test to the five-run F1 scores of the stochastic methods; for deterministic baselines, significance is assessed against the 95% confidence interval of TableManeuver, computed with the Student *t*-distribution.

The evaluation metrics are average precision (Avg P), average recall (Avg R), and the F1 score. Precision reflects the proportion of correctly predicted samples among those predicted to belong to a given maneuver class; recall reflects the proportion of correctly identified samples among those that truly belong to that class; and F1 is the harmonic mean of the two. These metrics assess the model from the perspectives of false alarms, missed detections, and overall recognition performance, respectively.

### 4.4. Main Results

Table 2 reports recognition performance on the F-18 test set. TableManeuver achieves the best results on all three metrics, with an average precision of 96.2% and an average recall of 96.8%. Compared with the strongest supervised baseline, ConvBiFormer, it improves precision by 5.4 percentage points, recall by 1.4 percentage points, and F1 by 3.5 percentage points. This improvement is statistically significant: Welch’s *t*-test on the five-run F1 scores yields *p* < 0.001 against both ConvBiFormer and TimeReasoner, and the 95% confidence interval of the TableManeuver F1 score, [96.3, 96.7], excludes the results of all baselines. Although MACTW requires no training, its distance-based discrimination cannot capture high-dimensional feature interactions or semantic-level maneuver patterns, leading to relatively low performance. The retrieval-only baselines quantify the contribution of nearest-neighbor label transfer alone. Moreover, 1-NN (DTW) label transfer and kNN (DTW, k=3) majority vote reach F1 scores of only 81.8% and 84.7%, respectively. These results show that DTW retrieval over the labeled library captures useful instance-level similarity, but copying neighbor labels without reasoning is insufficient. TableManeuver without neighborhood samples (a pure parameter-update-free LLM with no retrieval, F1 = 92.7%; Section 4.6.5) already exceeds the best retrieval-only baseline by 8.0 percentage points in F1. This result indicates that the LLM contributes substantive reasoning rather than merely propagating retrieved labels. The full model further improves on kNN by 11.8 points by combining reasoning with retrieval. The deep learning baselines improve through end-to-end learning, but some show imbalanced precision and recall. For example, LSTM reaches a recall of 91.5% but a precision of only 80.8%, indicating a tendency toward false alarms on easily confused categories.

Among the LLM-driven methods, Time-LLM loses information when reprogramming time series data into the language space, and its performance falls below that of some deep learning models. TimeReasoner improves on Time-LLM by guiding the LLM through chain-of-thought reasoning. However, it directly concatenates numerical sequences into text strings and does not preserve temporal-channel structure, which limits the model’s understanding of flight parameter sequences. In contrast, TableManeuver preserves temporal and channel structure through tabular encoding. It also combines domain knowledge and neighborhood samples through structured prompts, grounding LLM reasoning in semantic evidence rather than shallow pattern matching. The resulting reasoning trace provides an interpretable basis for the recognition decision, which is absent from discriminative baselines. Overall, TableManeuver achieves a favorable balance between recognition performance and interpretability.

To further analyze the recognition capability of the model on easily confused maneuvers, we compare TableManeuver with ConvBiFormer on four representative maneuver categories; the per-class results are shown in Table 3. For categories with prominent and geometrically simple features, such as aileron roll and loop, both methods achieve high recognition performance. For Split-S and Half Cuban Eight, which share similar motion segments, TableManeuver delivers more stable recognition. Specifically, on the Half Cuban Eight maneuver, the per-class precision and recall of TableManeuver are 7.6 and 7.0 percentage points higher than those of ConvBiFormer, respectively. These results indicate that domain knowledge and neighborhood samples can provide the LLM with maneuver definitions and temporal references, thereby giving the model a clearer discriminative basis when separating highly similar complex maneuvers.

### 4.5. Cross-Aircraft Generalization

To assess the generalization ability of the model in cross-domain scenarios, we evaluate it on the cross-aircraft F-16 test set. All baselines that require training are trained on the F-18 training set and then directly tested on the F-16 test set. TableManeuver uses neither labeled F-16 samples for training nor for retrieval; only the information about the target aircraft is updated in the prompt, while the F-18 training set continues to serve as the neighborhood-sample library.

Table 4 shows substantial differences in cross-aircraft generalization across methods. The deep learning baselines all degrade on the F-16 test set, with ConvBiFormer dropping by 6.6 percentage points in precision and 7.3 percentage points in recall. The retrieval-only baselines degrade more sharply. The kNN majority-vote F1 decreases from 84.7% on F-18 to 77.6% on F-16, indicating that nearest-neighbor label transfer is sensitive to aircraft-specific numerical distributions. In contrast, TableManeuver achieves precision and recall of 94.8% and 95.4% on the F-16 test set, with an F1 score only 1.4 percentage points below the F-18 result. This advantage is likewise statistically significant (Welch’s t-test on the five-run F1 scores, *p* < 0.001 against both ConvBiFormer and TimeReasoner). The gap between TableManeuver and the kNN baseline widens from 11.8 points on F-18 to 17.5 points on F-16. This provides further evidence that cross-aircraft robustness comes from LLM reasoning over shared domain knowledge rather than label transfer alone. These results suggest that tabular flight parameter representation and cross-aircraft domain knowledge reduce dependence on aircraft-specific numerical distributions and improve recognition stability.

### 4.6. Ablation Studies

To gain insight into the effectiveness of each key component of TableManeuver, a series of ablation experiments is performed.

#### 4.6.1. Effectiveness of Temporal and Channel Information

To assess the importance of the temporal and channel information preserved by the tabular encoding, three ablation experiments are conducted: removing temporal information by deleting the timestamps in the row indices; removing channel information by concatenating all parameter channels into a single sequence; and removing both. As shown in Table 5, both types of information contribute significantly to the model’s performance, with the temporal information having a more pronounced effect. The results indicate that temporal information plays a crucial role in helping LLMs understand the dynamic evolution of flight parameter sequences.

#### 4.6.2. Effectiveness of Contextual Information

To evaluate the effect of contextual information on FMR performance, we ablate the domain knowledge and context description components separately. The domain knowledge contains the standard definitions and discriminative features of each maneuver, whereas the context description covers dataset characteristics and the physical semantics of the channels. As shown in Table 6, removing the domain knowledge causes a drop of 4.1 and 4.2 percentage points in precision and recall, respectively, demonstrating that maneuver definitions are essential for the LLM to accurately recognize complex maneuvers. The impact of removing the context description is relatively smaller, leading to drops of 1.1 and 1.3 percentage points in precision and recall. These results indicate the joint importance of the semantic grounding provided by domain knowledge and the contextual constraints carried by the dataset description in supporting LLM reasoning; both are integral components of TableManeuver.

#### 4.6.3. Effectiveness of Task Decomposition

As shown in Table 6, removing the task decomposition mechanism leads to a clear performance drop, with precision and recall decreasing by 4.7 and 4.5 percentage points, respectively. This indicates that, without structured reasoning guidance, the LLM tends to produce an answer directly while overlooking key discriminative details. The experiment confirms that explicit task decomposition can effectively constrain the reasoning path and improve both the reliability and interpretability of complex discrimination tasks.

#### 4.6.4. Comparison of Tabular Encoding Formats

The choice of tabular encoding format has an important effect on the understanding and reasoning of LLMs. Different encoding methods produce different numbers of tokens, which directly affects how many neighborhood samples can fit into the context window. They also differ in how clearly they express the two-dimensional relationship between time steps and parameter channels, which in turn affects the accuracy of LLM reasoning.

To evaluate the impact of different tabular encoding formats on recognition performance, four representative encodings are compared: DFLoader, Markdown, HTML, and JSON. As shown in Table 7, DFLoader achieves the best performance. By aligning columns with whitespace and using no extra markup symbols, it strikes the best balance between token efficiency and structural clarity. Markdown ranks second; the use of separators and alignment lines introduces redundant tokens. HTML incurs even greater overhead due to nested tags and tends to distract reasoning. JSON expresses values per time step as key-value pairs, which fragments the overall temporal structure and yields the lowest performance. These results show that the token efficiency of the encoding and its ability to preserve the tabular structure are key factors influencing the reasoning performance of LLMs.

#### 4.6.5. Effectiveness of Neighborhood Samples

To verify the neighborhood-augmented reasoning mechanism, we compare three retrieval configurations: no neighborhood samples, positive samples only, and both positive and negative samples. As shown in Table 8, positive samples alone improve precision and recall by 2.0 and 1.9 percentage points, respectively, confirming the guiding role of nearest-neighbor references. Adding one negative sample further raises precision and recall to 96.2% and 96.8%, indicating that contrastive information helps the LLM establish clearer category boundaries. To isolate the contribution of negative sampling, we vary $k_{neg}\in \{0,1,2,3\}$ while fixing the number of positive samples at k=3 (lower part of Table 8). Performance peaks at $k_{neg}=1$ and then decreases monotonically, with F1 scores of 96.5%, 96.2%, and 95.9% for $k_{neg}=1,2,3$, respectively. This pattern suggests that one contrastive example provides the most useful boundary information, whereas additional negatives mainly add distracting context. As illustrated later in this subsection, under the heading ‘Comparison with horizontal maneuvers such as Steep turn’, the model explicitly cites the retrieved negative sample (‘Negative sample 1 shows that this kind of maneuver has roll angles steadily around 70 deg+, continuous heading change of 360 deg…’) to rule out steep-turn-like categories. This indicates that the negative sample is actively used in reasoning rather than merely included in the prompt.

### 4.7. Analysis of the Neighborhood Retrieval Strategy

#### 4.7.1. Choice of Distance Function

**Figure 6:**
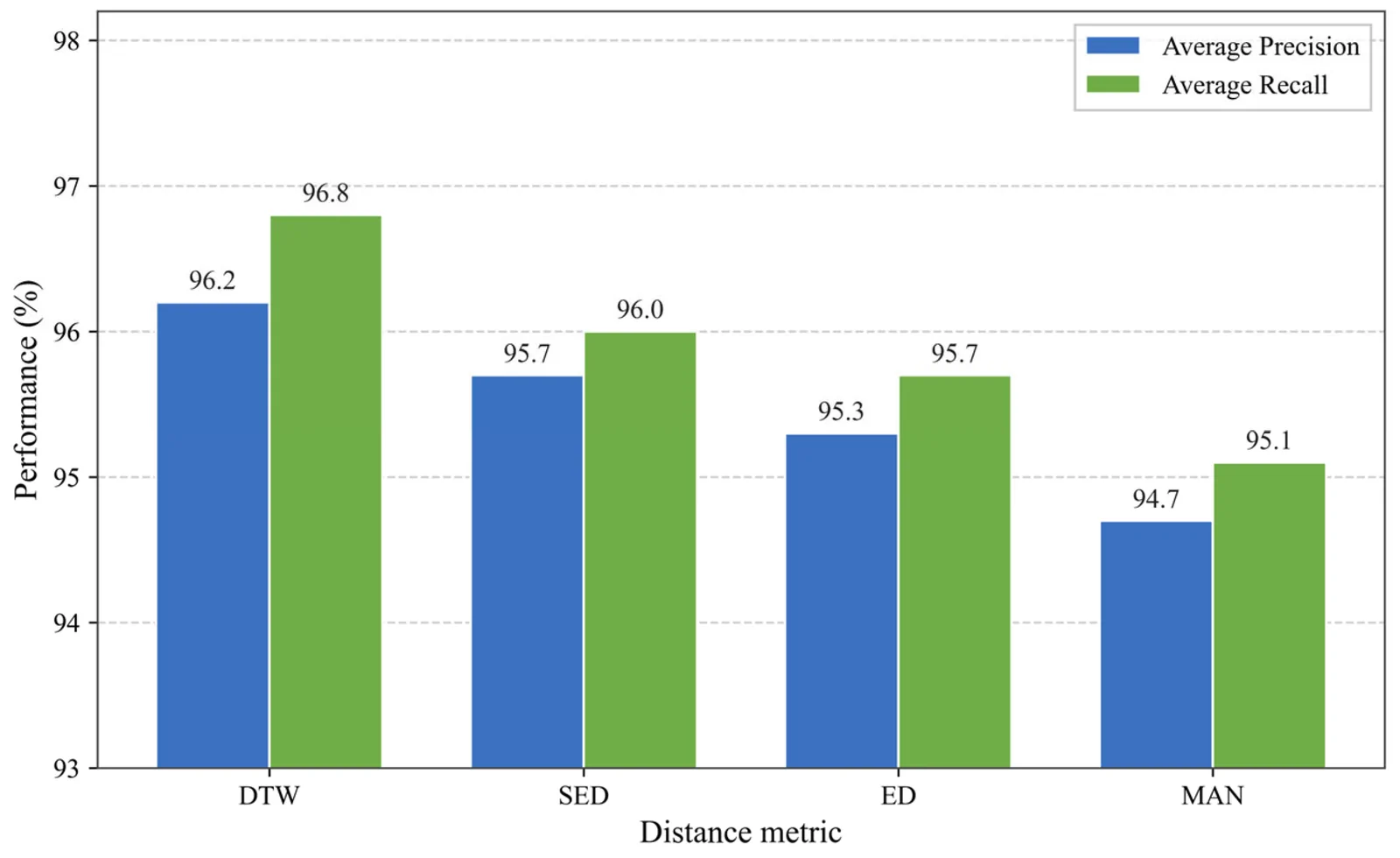
Performance comparison of four distance functions for neighborhood retrieval: DTW, SED, ED, MAN.

At the core of neighborhood retrieval lies the choice of similarity measure. We compare four representative distance functions: Dynamic Time Warping (DTW), Euclidean Distance (ED), Standardized Euclidean Distance (SED), and Manhattan Distance (MAN). The results are shown in Figure 6. As can be seen, DTW significantly outperforms the other three measures in both precision and recall. The reason is that DTW flexibly aligns temporal information through dynamic programming and can effectively accommodate the temporal variations and length differences in flight maneuver data. By contrast, ED and SED are based on point-to-point matching and are sensitive to temporal shifts; although MAN is somewhat more robust in high-dimensional spaces by avoiding the squared amplification, it still cannot handle temporal alignment. Considering both recognition accuracy and practical requirements, DTW is chosen as the default neighborhood retrieval distance function for TableManeuver.

#### 4.7.2. Effect of the Number of Neighborhood Samples

**Figure 7:**
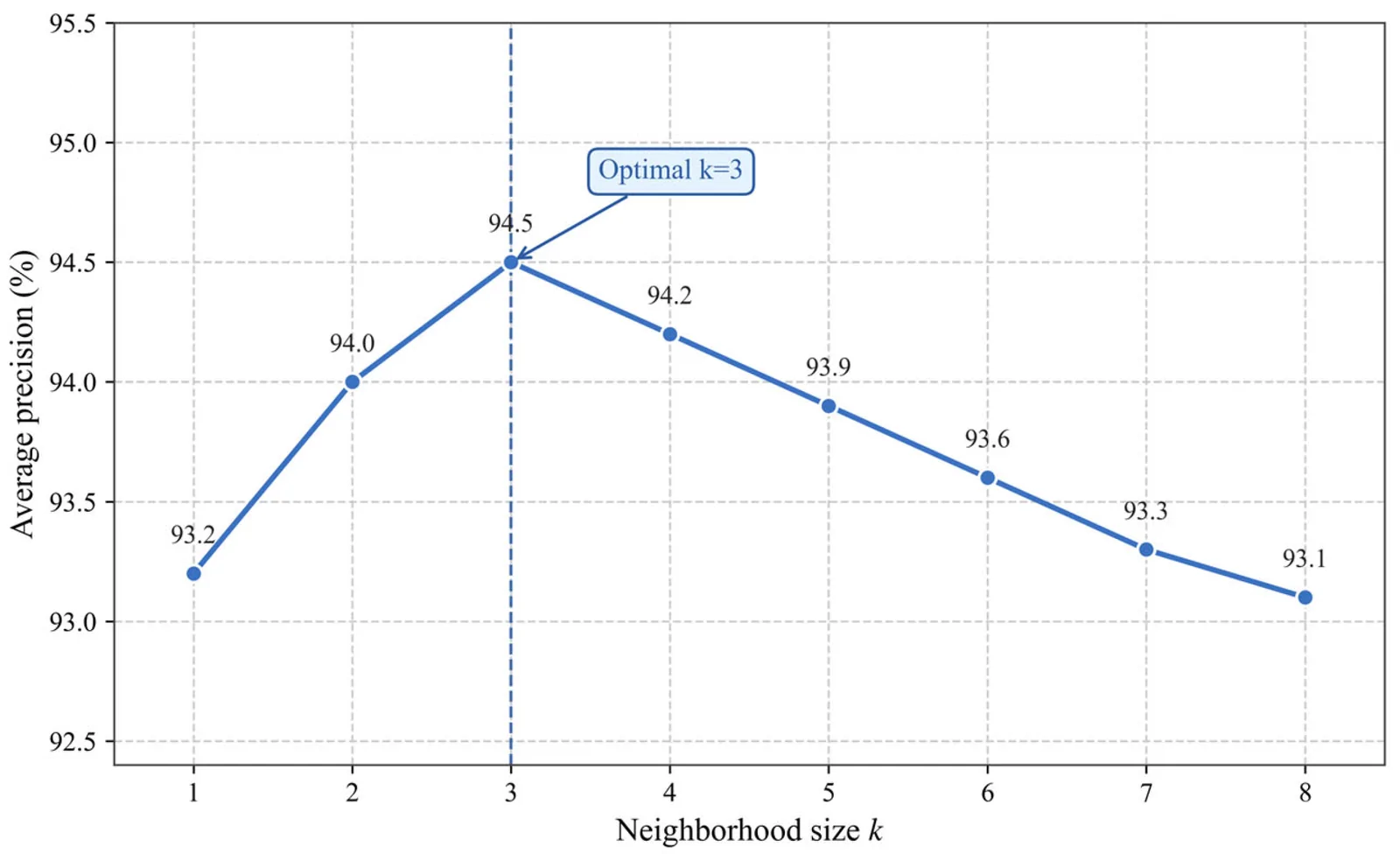
Precision under different neighborhood sample sizes.

To investigate the influence of the number of neighborhood samples on recognition performance, we vary the number of positive samples k without using negative samples. As shown in Figure 7, the precision first rises and then falls with k: it increases from 93.2% to a peak of 94.5% as k rises from 1 to 3, and then gradually drops to 93.1% when k further increases to 8. These observations suggest that a moderate number of positive samples improves performance by providing relevant context, but too many neighborhood samples may introduce noise or irrelevant patterns that hinder the model from focusing on key discriminative features. Moreover, an excessively large k markedly increases the input token count and the resulting computational cost. Balancing recognition performance and computational efficiency, we set k=3.

#### 4.7.3. Effect of Negative Samples

Introducing negative samples provides the LLM with counter-examples and guides it toward more comprehensive reasoning. The experiments show that, when only a single negative sample is provided, recognition accuracy initially decreases as the number of positive samples grows. A possible explanation for this counter-intuitive observation is that, when the number of positive samples is insufficient, the model cannot yet establish meaningful patterns or clear category boundaries, and the negative sample interferes with the initial learning of the positive-class pattern. However, as the number of positive samples further increases, recognition accuracy improves significantly and ultimately surpasses the best performance achieved with positive samples alone. This suggests that the LLM benefits from richer and more diverse positive representations, and that a sufficient number of positive samples is a necessary foundation for the contrastive role of negative samples to take effect.

### 4.8. Performance Comparison Across Base LLMs

Considering that different base LLMs differ in reasoning capability, this section further analyzes the performance of several representative LLMs combined with the TableManeuver framework. The selected models include the closed-source high-performance models Gemini-3.1 Pro and GPT-5.4, the open-source full-parameter model deepseek-v4-pro, and the open-weight models Qwen3.6-35B-A3B and Qwen3.5-4B. All models share the same tabular encoding, task prompt, generation temperature, and output parsing rules.

The results are reported in Table 9. As can be seen, all base LLMs achieve precision and recall above 90% under the TableManeuver framework. Specifically, the closed-source models Gemini-3.1 Pro and GPT-5.4 yield the best performance, with both precision and recall exceeding 96%. The open-source full-parameter model deepseek-v4-pro follows closely. Qwen3.6-35B-A3B offers a favorable balance between open-source availability and recognition accuracy. The lightweight open-weight model Qwen3.5-4B, owing to its smaller parameter scale, is slightly weaker but still clearly outperforms most supervised deep learning baselines, showing that the structured prompting strategy remains effective at relatively small model scales. In summary, the core mechanisms of TableManeuver do not rely on any specific base model and can be transferred across different LLM families. Considering both performance and availability, all remaining experiments in this paper are conducted with Qwen3.6-35B-A3B.

We emphasize that the prompt was not re-tuned for each model: all five models in Table 9 receive the identical prompt, i.e., the same domain context, neighborhood context, tabular serialization, and task decomposition instruction, with only engineering-level differences in API message wrapping and maximum output length. The F1 spread among the four larger models is within 0.8 percentage points, indicating that the prompt transfers directly across LLM families without model-specific redesign. The degradation of the lightweight Qwen3.5-4B stems from model capacity rather than prompt mismatch, as its errors concentrate on the same confusable categories. In practice, minor adaptations of the output-format instruction may be needed for models with weaker instruction-following ability, but the prompt content itself requires no modification.

### 4.9. Case Study

**Figure 8:**
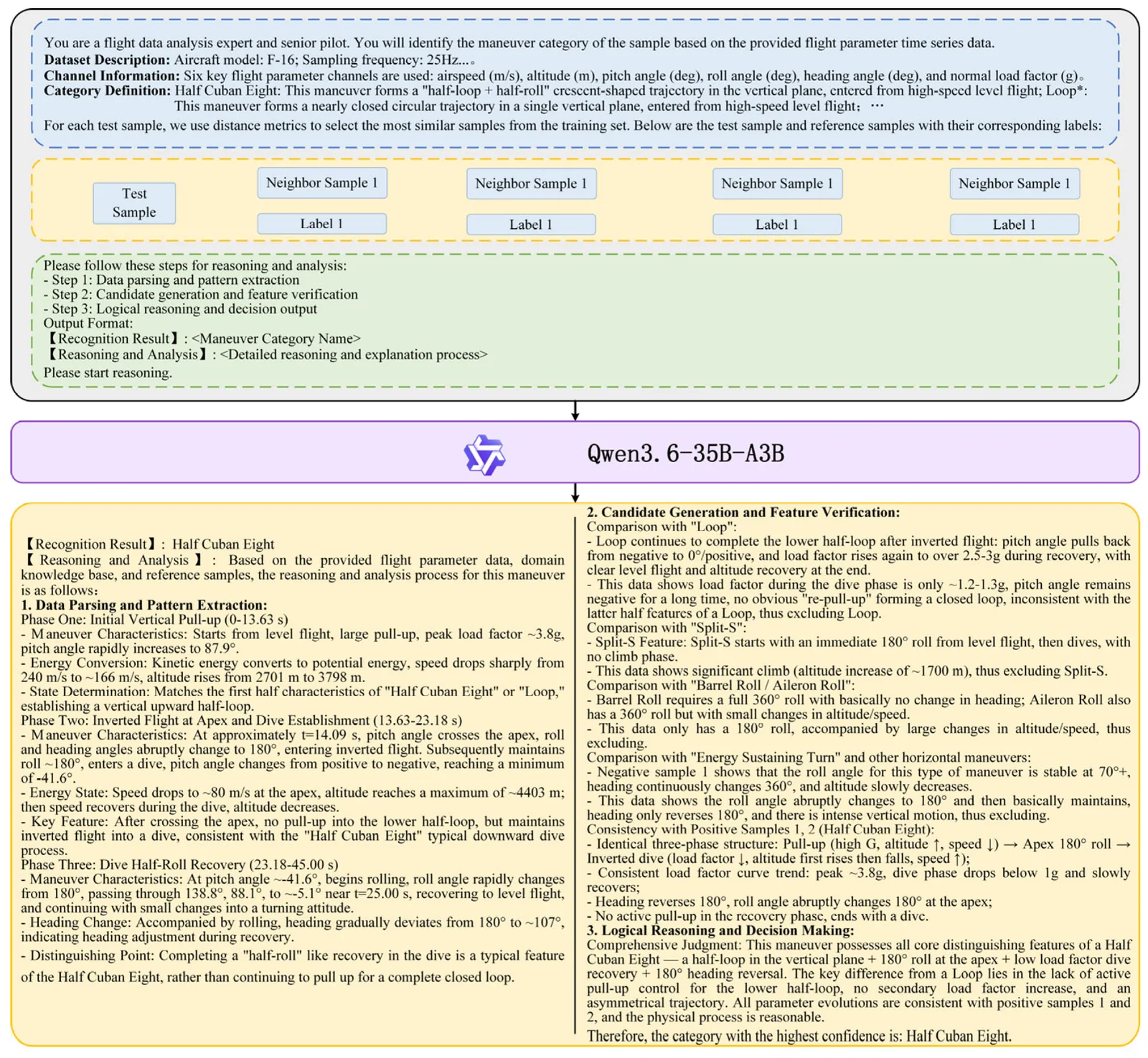
Case study of TableManeuver identifying a Half Cuban Eight maneuver: input prompt, model reasoning trace, and output.

To intuitively illustrate the interpretable reasoning process of TableManeuver, Figure 8 presents a complete case in which a Half Cuban Eight maneuver is correctly recognized. The case clearly shows that the recognition decision of TableManeuver is not a simple pattern match but an explicit, phase-wise reasoning grounded in flight domain knowledge, and that its interpretability is markedly superior to that of discriminative black-box models.

### 4.10. Human Evaluation of Interpretability

To systematically verify the quality of the reasoning traces generated by TableManeuver, we adopt a multi-dimensional human evaluation protocol [34]. Fifty correctly recognized samples are randomly drawn from the F-18 test set, and their reasoning traces are submitted to three senior flight instructors—each with more than five years of flight instruction experience—for independent blind evaluation. The evaluation covers four dimensions, each rated on a 1–5 scale: (1) numerical consistency assesses whether the numerical evidence cited in the reasoning is consistent with the raw flight parameters; (2) logical correctness assesses whether the reasoning chain is rigorous and the causal inferences are sound; (3) reasoning completeness assesses whether all key phases of the maneuver are covered; and (4) readability assesses whether the textual structure is clear and the terminology is accurate. Inter-rater consistency is measured by the Intraclass Correlation Coefficient (ICC). The results are reported in Table 10.

The inter-rater ICC is 0.83, which indicates a good level of agreement and confirms the reliability of the evaluation. As shown in Table 10, TableManeuver receives high scores in all four dimensions, with an overall mean of 4.49. Readability obtains the highest score of 4.61, mainly because the task decomposition mechanism organizes the reasoning into a clear three-step structure that yields well-layered output text. The numerical-consistency score of 4.52 indicates that the vast majority of reasoning traces accurately cite numerical evidence from the flight parameter data. The logical-correctness score is slightly lower at 4.38; the lost points are mostly concentrated in the comparative reasoning between easily confused maneuvers, pointing to a specific weakness to be strengthened in future work.

### 4.11. Failure Case Analysis

To better understand the limitations of the framework, we examine all misclassified samples of the F-18 test set (13 out of 360) and group the failures into three types.

(1) Confusion between structurally overlapping composites (9 of 13 errors). Most misclassifications occur between Split-S and Half Cuban Eight when the initial climb segment of the Half Cuban Eight is shallow. In these cases, the discriminative half-loop preceding the inverted dive is weakly expressed in the data itself, and the reasoning trace shows that the model correctly extracts the phase structure but assigns the borderline climb evidence to the wrong candidate.

(2) Retrieval-induced errors (3 of 13 errors). When all k positive neighbors returned by DTW retrieval belong to a wrong but similar category, the LLM anchors its comparison on the mislabeled references and inherits the error. These cases indicate that retrieval quality upper-bounds the benefit of neighborhood augmentation, and a label consistency check over the retrieved set is a straightforward safeguard.

(3) Reasoning inconsistency (1 of 13 errors). In rare cases the trace cites correct numerical evidence for each phase but draws an unsupported conclusion in the final elimination step. This failure mode is consistent with the slightly lower logical-correctness score observed in the human evaluation (Section 4.10) and motivates automatic consistency checking between the cited evidence and the final decision.

These observations suggest three concrete improvements, which we leave for future work: finer-grained phase definitions in the domain knowledge for confusable composites, label-aware retrieval safeguards, and automatic output consistency verification.

## 5. Conclusions

To address the issues that existing flight maneuver recognition methods rely on large-scale labeled data, have limited cross-aircraft adaptability, and lack transparency in their decisions, this paper reformulates FMR as a table-understanding task and proposes TableManeuver, a tuning-free explainable recognition method based on LLMs. The proposed method converts flight parameter time series into a structured textual representation via tabular encoding, provides instance-level context through neighborhood retrieval, and guides the LLM to perform explicit step-by-step reasoning through structured prompts. Experiments on a high-fidelity simulated flight dataset show that, compared with strong supervised baselines, the proposed method achieves the best F1 score of 96.5%, suffers only a 1.4 percentage point drop in F1 on the cross-aircraft test set, and produces reasoning traces that are rated highly by domain experts for interpretability. The method handles complex flight maneuver recognition tasks well. Future work will focus on validation with real flight data, and will explore acceleration mechanisms such as approximate retrieval, reasoning caching, and model distillation, while extending the framework to tasks such as flight quality assessment, flight intent recognition, and anomaly detection.

## Figures and Tables

**Table 1:** Statistical information of the constructed flight maneuver dataset.

Subset	Aircraft	Categories	Pilots	Samples	Sequence Length (Frames)	Mean Length (Frames/s)
Training set	F-18	9	16	1440	426–2810	1165/116.5
Test set (F-18)	F-18	9	4	360	438–2756	1196/119.6
Test set (F-16)	F-16	9	5	450	512–3024	1328/132.8

**Table 2:** Performance comparison between TableManeuver and baseline methods on the F-18 test set.

Method	Paradigm	Training Mode	Avg *P* (%)	Avg R (%)	F1 (%)
MACTW	Traditional	Tuning-free	76.8	82.1	79.4
1-NN (DTW)	Retrieval-only	Tuning-free	81.5	82.1	81.8
kNN (DTW, k = 3)	Retrieval-only	Tuning-free	84.4	85.0	84.7
CNN	Deep learning	Supervised	89.5 ± 0.3	94.2 ± 0.5	91.8 ± 0.2
LSTM	Deep learning	Supervised	80.8 ± 0.4	91.5 ± 0.2	85.8 ± 0.3
ConvBiFormer	Deep learning	Supervised	90.8 ± 0.4	95.4 ± 0.3	93.0 ± 0.5
Time-LLM	LLM-driven	Fine-tuned	82.5 ± 0.2	85.3 ± 0.4	83.9 ± 0.2
TimeReasoner	LLM-driven	Tuning-free	88.1 ± 0.3	90.5 ± 0.4	89.3 ± 0.3
TableManeuver (ours)	LLM-driven	Tuning-free	96.2 ± 0.3	96.8 ± 0.3	96.5 ± 0.2

**Table 3:** Performance comparison between TableManeuver and ConvBiFormer on selected maneuver categories.

Maneuver	Characteristics	Model	*p* (%)	R (%)	F1 (%)
Aileron roll	Single motion pattern; highly distinguishable temporal features	ConvBiFormer	97.8	98.2	98.0
		TableManeuver	99.3	99.1	99.2
Loop	Standard vertical maneuver with a clear profile	ConvBiFormer	96.1	96.8	96.4
		TableManeuver	98.7	98.5	98.6
Split-S	Shares segments with dive and half-loop	ConvBiFormer	88.5	89.3	88.9
		TableManeuver	94.9	95.8	95.3
Half Cuban Eight	Composite maneuver combining half-loop, roll, and dive; complex and easily confused	ConvBiFormer	86.2	87.6	86.9
		TableManeuver	93.8	94.6	94.2

**Table 4:** Cross-aircraft generalization performance comparison on the F-16 test set.

Method	Training Mode	Avg *p* (%)	Avg R (%)	F1 (%)
MACTW	Tuning-free	72.5	78.3	75.3
1-NN (DTW)	Tuning-free	74.5	75.1	74.8
kNN (DTW, k = 3)	Tuning-free	77.2	78.0	77.6
CNN	Supervised	68.0 ± 0.3	84.3 ± 0.5	75.3 ± 0.2
LSTM	Supervised	65.2 ± 0.4	80.1 ± 0.2	71.9 ± 0.3
ConvBiFormer	Supervised	84.2 ± 0.4	88.1 ± 0.3	86.1 ± 0.5
Time-LLM	Fine-tuned	75.8 ± 0.2	79.2 ± 0.4	77.5 ± 0.2
TimeReasoner	Tuning-free	86.5 ± 0.3	88.3 ± 0.4	87.4 ± 0.3
TableManeuver (ours)	Tuning-free	94.8 ± 0.4	95.4 ± 0.3	95.1 ± 0.3

**Table 5:** Ablation study on temporal and channel information.

Setting	Avg *p* (%)	Avg R (%)	Δ (%)
w/all information (full model)	96.2 ± 0.3	96.8 ± 0.3	—
w/o temporal information	93.5 ± 0.4	93.8 ± 0.3	−2.7/−3.0
w/o channel information	94.2 ± 0.5	94.5 ± 0.2	−2.0/−2.3
w/o temporal & channel information	91.5 ± 0.4	91.8 ± 0.2	−4.7/−5.0

Note: “w/” denotes “with” and “w/o” denotes “without”; all experiments are conducted on the F-18 test set.

**Table 6:** Ablation study on prompt components.

Setting	Avg *p* (%)	Avg R (%)	Δ (%)
w/all information (full model)	96.2 ± 0.3	96.8 ± 0.3	—
w/o domain knowledge	92.1 ± 0.4	92.6 ± 0.3	−4.1/−4.2
w/o context description	95.1 ± 0.5	95.5 ± 0.2	−1.1/−1.3
w/o domain knowledge & context description	91.0 ± 0.4	91.3 ± 0.2	−5.2/−5.5
w/o task decomposition	91.5 ± 0.3	92.3 ± 0.4	−4.7/−4.5

**Table 7:** Performance comparison of different table encoding formats on the F-18 test set.

Format	Avg *p* (%)	Avg R (%)	Δ (%)
DFLoader	96.2 ± 0.3	96.8 ± 0.3	—
Markdown	95.6 ± 0.4	96.0 ± 0.3	−0.6/−0.8
HTML	95.3 ± 0.5	95.8 ± 0.2	−0.9/−1.0
JSON	94.9 ± 0.4	95.5 ± 0.2	−1.3/−1.3

**Table 8:** Ablation study on neighborhood retrieval strategies.

Retrieval Strategy	Avg *p* (%)	Avg R (%)	Δ (%)
w/o neighborhood samples (pure tuning-free inference)	92.5± 0.2	92.9± 0.3	−3.7/−3.9
w/positive samples only (k=3$,\;k_{neg}=0$)	94.5± 0.4	94.8± 0.2	−1.7/−2.0
w/positive and negative samples (k=3$,\;k_{neg}=1$)	96.2 ± 0.3	96.8 ± 0.3	—
w/positive and negative samples (k=3$,\;k_{neg}=2$)	96.0 ± 0.2	96.5 ± 0.3	−0.2/−0.3
w/positive and negative samples (k=3$,\;k_{neg}=3$)	95.7 ± 0.1	96.1 ± 0.3	−0.5/−0.7

**Table 9:** Performance comparison of TableManeuver across different base LLMs.

Model	Type	*p* (%)	R (%)	F1 (%)
Gemini-3.1 Pro	Closed-source, high-performance	97.1 ± 0.2	97.3 ± 0.3	97.2 ± 0.4
GPT-5.4	Closed-source, high-performance	96.9 ± 0.3	97.1 ± 0.5	97.0 ± 0.2
deepseek-v4-pro	Open-source, full-parameter	96.3 ± 0.4	96.6 ± 0.2	96.4 ± 0.3
Qwen3.6-35B-A3B	Open-weight	96.2 ± 0.3	96.8 ± 0.3	96.5 ± 0.2
Qwen3.5-4B	Open-weight (lightweight)	92.4 ± 0.2	92.9 ± 0.4	92.6 ± 0.2

**Table 10:** Human evaluation results of reasoning trace quality (1–5 scale).

Dimension	Mean	Std. Dev.	Min	Max
Numerical consistency	4.52	0.41	3.5	5.0
Logical correctness	4.38	0.48	3.0	5.0
Reasoning completeness	4.45	0.44	3.5	5.0
Readability	4.61	0.37	4.0	5.0
Overall average	4.49	0.43	—	—

## Data Availability

The code is publicly available at https://github.com/BH0001/TableManeuver (accessed on 8 July 2026). Requests to access the dataset should be directed to the corresponding author.

## Dual-Use Statement

Current research is limited to the field of flight maneuver recognition for flight training assessment and flight safety monitoring, which is beneficial for improving the quality of flight training, supporting human–machine cooperative flight, and enhancing aviation safety, and does not pose a threat to public health or national security. The authors acknowledge the dual-use potential of the research involving flight parameter time series analysis and confirm that all necessary precautions have been taken to prevent potential misuse. As an ethical responsibility, the authors strictly adhere to relevant national and international laws about Dual-Use Research of Concern (DURC). The authors advocate for responsible deployment, ethical considerations, regulatory compliance, and transparent reporting to mitigate misuse risks and foster beneficial outcomes.
